# Emerging roles of Axin in cerebral cortical development

**DOI:** 10.3389/fncel.2015.00217

**Published:** 2015-06-08

**Authors:** Tao Ye, Amy K. Y. Fu, Nancy Y. Ip

**Affiliations:** Division of Life Science, Molecular Neuroscience Center and State Key Laboratory of Molecular Neuroscience, The Hong Kong University of Science and TechnologyHong Kong, China

**Keywords:** Axin, cerebral cortex, neurogenesis, polarization, axon formation, cytoskeletal regulation

## Abstract

Proper functioning of the cerebral cortex depends on the appropriate production and positioning of neurons, establishment of axon–dendrite polarity, and formation of proper neuronal connectivity. Deficits in any of these processes greatly impair neural functions and are associated with various human neurodevelopmental disorders including microcephaly, cortical heterotopias, and autism. The application of *in vivo* manipulation techniques such as *in utero* electroporation has resulted in significant advances in our understanding of the cellular and molecular mechanisms that underlie neural development *in vivo*. Axin is a scaffold protein that regulates neuronal differentiation and morphogenesis *in vitro*. Recent studies provide novel insights into the emerging roles of Axin in gene expression and cytoskeletal regulation during neurogenesis, neuronal polarization, and axon formation. This review summarizes current knowledge on Axin as a key molecular controller of cerebral cortical development.

## Introduction

The mammalian cerebral cortex is characterized by a six-layered laminar structure, which forms the structural basis for higher cognitive function. Each cortical layer contains distinct distributions of neuron types with specific dendritic morphology, electrophysiological properties, and axonal connection with other brain regions. Remarkably, the characteristic distribution and connectivity of cortical neurons originate from a single layer of progenitor cells called the neuroepithelium. During cortical development, neural progenitor cells located in the ventricular zone undergo symmetric cell division to proliferate and maintain a proper progenitor pool; moreover, they utilize asymmetric cell division to differentiate and generate successive waves of neurons. After neuronal differentiation, the postmitotic neurons go through a multipolar stage; they subsequently polarize, taking on a bipolar morphology with a leading process towards the cortical plate and a nascent axon toward the opposite direction. The cell body continues to migrate along radial glial fibers towards the cortical plate and past the existing layers of neurons. Thus, cortical layers are created in an inside-out manner, with the early- and later-born neurons occupying the deeper and superficial layers, respectively.

When the neurons mature at their destination, the leading process spawns the apical dendrite, followed by dendritic arborization and synaptic formation. The nascent axon elongates tangentially, projecting to form synaptic connections with other neurons. Defects in any of these coordinated events can greatly impair neural functions and are implicated in various neurological and psychiatric disorders including lissencephaly, cortical heterotopias, and autism (Ayala et al., 2007). Thus, these steps require the orchestration of various signaling cascades; accordingly, scaffold proteins have emerged as key molecular controls of cortical development owing to their abilities to interact with and regulate a myriad of signaling proteins. This review summarizes the emerging roles of the scaffold protein Axin in the orchestration of the transcriptional program of neurogenesis and cytoskeletal regulation of neural development in the cerebral cortex.

## Identification and Regulation of Axin

Axin was initially identified from the analysis of the *Fused* locus, mutations of which cause defects in axis formation in mouse embryos (Zeng et al., 1997). Because of its inhibition of axis formation, the *Fused* gene was named *Axis inhibitor* (*Axin*). Genetic analyses identified Axin as a negative regulator of the canonical Wnt signaling pathway, although its precise roles remained unclear (Kikuchi, 1999). Biochemical studies demonstrated that Axin acts as a scaffold and associates with various components of the canonical Wnt signaling pathway, forming the β-catenin destruction complex, which leads to the GSK3β–dependent phosphorylation of β-catenin (Luo and Lin, 2004).

**Figure 1:**
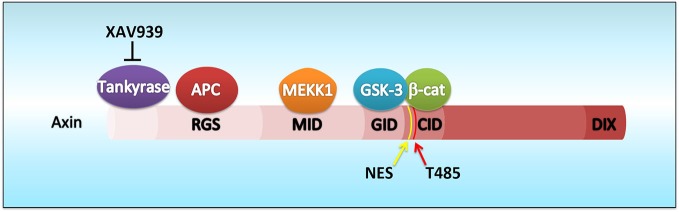
**Schematic diagram of Axin domains, molecular interactions and Cdk5-dependent phosphorylation site**. Axin possesses functional domains including the Regulators of G protein signaling (RGS which interacts with adenomatosis polyposis coli, APC) and DIX domains. Axin contains domains for interacting with other proteins such as MEKK1 (MID), GSK3β (GID), and β-catenin (CID). A small molecule, XAV939, stabilizes Axin by inhibiting the poly-ADP-ribosylating enzyme tankyrase which stimulates Axin degradation through the ubiquitin-proteasome pathway. It is worth noting that the Cdk5-dependent phosphorylation site (Thr485) is located close to the nuclear export signal (NES) of Axin (amino acids 413–423).

Axin possesses several functional domains including the Regulators of G protein signaling domain near its N-terminus and the C-terminal DIX domain, which is also found in Disheveled and Dixdc1 (also called Ccd1). In addition, Axin contains domains for interacting with other proteins such as GSK3β and β-catenin (Figure 1). In brief, Axin interacts with adenomatosis polyposis coli (APC), GSK3β, and β-catenin via distinct domains to form the β-catenin destruction complex (Furuhashi et al., 2001). Without Wnt ligand stimulation, Axin facilitates GSK3β-mediated phosphorylation of β-catenin, and thus triggers the proteasome-dependent ubiquitination and degradation of β-catenin. Hence, although β-catenin is constitutively expressed in the cytoplasm, its protein level remains relatively low because of destruction complex activity. When the Wnt ligands bind to the receptors Frizzled and LRP5/6, they activate several signaling components such as Disheveled. Disheveled associates with Axin, translocating Axin to the membrane, which leads to Axin degradation. Loss of Axin results in the disassembly of the destruction complex as well as the suppression of β-catenin phosphorylation by GSK3β. Subsequently, β-catenin accumulates and translocates into the nucleus, where it forms a complex with TCF/Lef transcription factors and turns on Wnt-responsive gene transcription (Furuhashi et al., 2001). It should be noted that most of these findings are from studies using different types of cultured cells. Thus, the precise mechanisms *in vivo* are only beginning to be elucidated.

Axin has also emerged as a master scaffold for multiple signaling pathways (Kikuchi, 1999). Axin interacts with and activates MEKK, which activates JNK, suggesting that Axin regulates the JNK pathway (Liu et al., 2006). Axin also functions as a scaffold in the transforming growth factor β (TGF-β) pathway by facilitating the ubiquitin E3 ligase Arkadia-dependent degradation of Smad7 (Zeng et al., 1997). It also directly interacts with Smad3 and promotes its phosphorylation by TGF-β receptors (Furuhashi et al., 2001; Guo et al., 2008).

## Coordination of Neurogenesis by Axin-Mediated Signaling

Neurons are generated from neural progenitor cells. Cortical neural progenitors include neuroepithelial progenitors, radial glial progenitors (RGPs; Ever and Gaiano, 2005), and intermediate progenitors (IPs; Farkas and Huttner, 2008). Among them, RGPs in the ventricular zone are responsible for generating most or all neurons during embryonic development (Kriegstein et al., 2002; Götz et al., 2003; Heintz et al., 2004). RGPs enlarge the progenitor pool through symmetric proliferative divisions in order to enable the subsequent rapid increase in neurons (Götz and Huttner, 2005; Huttner and Kosodo, 2005). As development proceeds, RGPs switch to neuronal differentiation, dividing asymmetrically and producing several neurons. RGPs adopt two modes of asymmetric divisions to generate neurons: producing one RGP for self-renewal and either one neuron (direct neurogenesis) or one neurogenic IP (indirect neurogenesis) to enlarge the population of neurons (Noctor et al., 2004; Götz and Huttner, 2005). Consequently, asymmetric cell division of RGPs plays a critical role in generating a proper number of neurons, while at the same time maintaining an adequate pool of neural progenitors for self-renewal. IPs that preferentially reside in the subventricular zone are transient neurogenic progenitors with limited amplifying capability (1–3 mitotic cycles). IPs undergo symmetric divisions to produce pairs of IPs that subsequently differentiate into neurons (Pontious et al., 2008; Kowalczyk et al., 2009). Thus, the birthdates and numbers of neurons are dependent on the balance between neural progenitor proliferation and differentiation.

**Figure 2:**
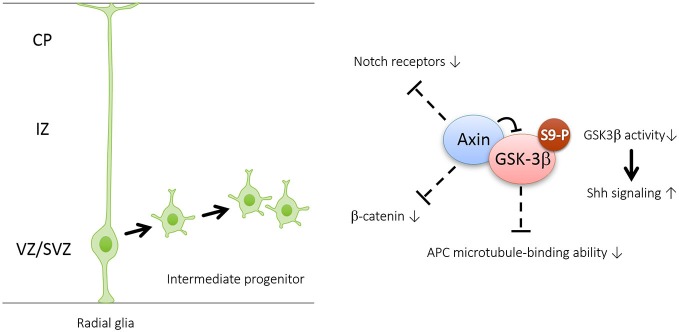
**Axin promotes the generation and amplification of intermediate progenitors by interacting with GSK3β in the cytoplasm**. Axin is well-characterized as a “master” scaffold for various signaling proteins including GSK3β, β-catenin, Notch and adenomatosis polyposis coli (APC)—all of which are known to control neurogenesis. Interaction with Axin can cause increased Ser9 phosphorylation and subsequent inhibition of GSK3β in the cytoplasm. GSK3β inhibition may enhance intermediate progenitor amplification by activating Shh signaling and repressing the expression levels of Notch receptors and β-catenin, as well as APC-regulated division plane.

**Figure 3:**
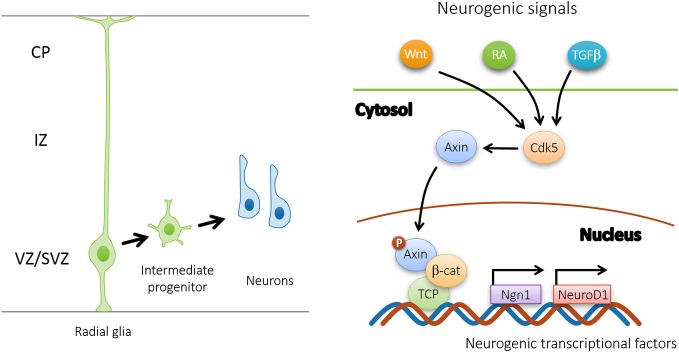
**Upon Cdk5 phosphorylation, Axin promotes the differentiation of intermediate progenitors by interacting with β-catenin in the nucleus**. In response to neurogenic signals (Wnt, RA, TGFβ, etc.), nuclear Axin forms complex with β-catenin and TCP to activate downstream neurogenic transcriptional factors, such as Ngn1 and NeuroD1.

Recent evidence suggests that Axin serves as a master scaffold for coordinating the proliferation and differentiation of neural progenitors during cerebral cortical development (Fang et al., 2013). Our laboratory has demonstrated that the level and subcellular localization of Axin in neural progenitors determine their fate, either self-proliferation or neuronal differentiation. In the presence of proliferating cues such as SHH, the Axin-GSK3β interaction in the cytoplasm is crucial for the proliferation of the intermediate progenitor cells (Figure 2). Upon stimulation by neurogenic cues such as WNT, RA, and TGFβ, the Axin-β-catenin interaction in the nucleus promotes neuronal production through the activation of neurogenic transcription factors (Figure 3). Of note, Axin phosphorylation at Thr485 (Figure 1) by cyclin-dependent kinase-5 (Cdk5) determines the subcellular localization of Axin, which translocates from the cytoplasm to the nucleus upon neural progenitor differentiation, thus serving as a molecular switch that causes IPs to switch from proliferation to differentiation (Fang et al., 2013).

## Axin-GSK3β Signaling in Neuronal Polarization and Migration

Newborn neurons exit the ventricular and subventricular zones, and migrate into the intermediate zone. They initially exhibit a multipolar morphology characterized by several thin processes in various directions but subsequently adopt a bipolar morphology with a leading process pointing toward the apical pia and a trailing process extending toward the ventricle. Directed by their leading processes, neurons migrate toward the cortical (i.e., pial) surface in an orderly progression to occupy their proper positions (Tsai and Gleeson, 2005). Each wave of migrating neurons travels past their predecessors, forming cortical layers of the cortical plate in an inside-out manner, in which the older and younger neurons reside in deeper layers closer to the ventricle and outer layers of the cerebral cortex closer to the pia, respectively (Noctor et al., 2004). Projection neurons generally migrate in two modes: radial glial fiber-independent somal translocation and radial glial fiber-dependent locomotion. Somal translocating neurons attach their long leading processes to the pia through Reelin-mediated cell-matrix adhesion (Sekine et al., 2012) and subsequently shorten them to move their cell bodies to their final positions and detach from the pia to terminate migration (Dulabon et al., 2000). Notably, as the cortical plate is sufficiently thin during the early stage of cortical development, early-generated neurons can extend leading processes to the pia and migrate through somal translocation alone (Nadarajah et al., 2001). On the other hand, locomoting neurons adhere to the radial glial fibers and extend their short leading processes to wrap around the fibers, forming a temporary adhesion that facilitates their migration along the fiber (Noctor et al., 2004; Ayala et al., 2007). Thus, various factors including the polarity and morphology of migrating neurons as well as adhesion with radial glial fibers contribute to the appropriate laminar positioning of neurons.

The first line of evidence suggesting that Axin plays a role in neuronal migration came from the effect of Axin overexpression in migrating neurons (Fang et al., 2013). Notably, a substantial number of Axin-overexpressing neurons are stacked in the intermediate zone, suggesting that Axin has an alternative function in neuronal migration probably through the regulation of GSK3 signaling (Fang et al., 2013). A recent study using mutant mice lacking both GSK3α and GSK3β confirms the *in vivo* role of GSK3 signaling in neuronal migration (Morgan-Smith et al., 2014). Conditional *Gsk3* deletion in cortical neurons under neuron-specific *Neurod6* promoter resulted in dramatic mislocalization of layer 2/3 neurons in deeper layers. In particular, *Gsk3* deletion disrupts the transition of cortical neurons from multipolar migration phase to bipolar migration phase (Morgan-Smith et al., 2014). Since GSK3β but not GSK3α interacts with Axin during neural development (Fang et al., 2011, 2013), these findings collectively suggest that Axin-GSK3β interaction can target the neuronal polarization process to regulate neuronal migration during cerebral cortical development.

How does Axin-GSK3β signaling specify the neuronal polarity? Increasing evidence suggests that GSK3β functions as a critical regulator of neuronal polarization and migration by controlling microtubule dynamics (Hur and Zhou, 2010). Initial studies on neuronal polarization came from cultured dissociated hippocampal neurons. Before polarity establishment, Ser9-phosphorylated GSK3β, which is the inactive form, is universally expressed at the tip of each neurite. Upon neuronal polarization, one of the neurites gives rise to the axon, where phosphorylated GSK3β becomes enriched at the tip of the nascent axon. Therefore, axon specification depends on the local inhibition of GSK3β in one neurite and activation of GSK3β in other neurites. Accordingly, multiple axons are induced when GSK3β activity is globally suppressed by pharmaceutical inhibitors or GSK3β-specific knockdown. In contrast, axon specification is impaired when the constitutive GSK3β-Ser9Ala mutant is overexpressed, and thus neuronal polarization is inhibited. Besides, the instructive role of GSK3β inactivation in axon specification is further demonstrated by the observation that the differentiated dendrite can be transformed into axons after axon-dendrite polarity specification. This evidence collectively indicates that local inhibition of GSK3β is essential for the establishment and maintenance of neuronal polarity (Hur and Zhou, 2010). Notably, we have suggested that the specific Axin-GSK3β interaction ensures the precise localization and inactivation of GSK3β in one neurite during neuronal polarization (Fang et al., 2011).

**Figure 4:**
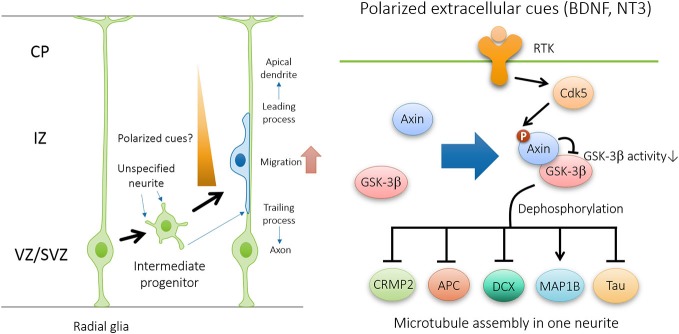
**Axin-GSK3β signaling regulates neuronal polarization during neuronal migration**. In the presence of polarized extracellular cues such as brain-derived neurotrophic factor (BDNF) and NT3, Cdk5-dependent phosphorylation of Axin facilitates Axin-GSK3β interaction and local inhibition of GSK3β activity at the tip of nascent axon, and thus promotes microtubule assembly through the regulation of various microtubule-associated proteins, including CRMP2, APC, DCX, MAP1B, and Tau.

Cytoskeletal reorganization is required for neurons to undergo morphological changes during polarization and migration. Several substrates of GSK3β have been identified as important downstream effectors in regulating neuronal polarization through microtubule rearrangement (Figure 4). These substrates includes microtubule plus-end binding proteins CRMP-2 (Yoshimura et al., 2005), APC (Zumbrunn et al., 2001), and doublecortin DCX (Bilimoria et al., 2010), as well as structural microtubule associated proteins (MAPs) such as MAP1B (Trivedi et al., 2005) and Tau (Johnson and Stoothoff, 2004). In polarizing neurons, plus-end binding proteins CRMP-2 and APC localizes at the tips of immature neurites and later becomes concentrated in the growth cone of the nascent axon (Inagaki et al., 2001; Zumbrunn et al., 2001). Binding of unphosphorylated active forms of CRMP2, DCX, APC, and Tau to microtubules promotes their assembly and stabilization. The microtubule-binding activity of these proteins is abrogated by GSK3β phosphorylation (Hur and Zhou, 2010). Therefore, GSK3β inactivation in the growth cone of the nascent axon facilitates the interaction between MAPs and microtubules, and thus promotes axon initiation by specifically stabilizing one neurite. Similar to the effect of global GSK3β inhibition, overexpression of phosphorylation-deficient CRMP2-Ser514Ala is sufficient to induce multiple axons during neuronal polarization. In addition, GSK3β-mediated phosphorylation of MAP1B may also contribute to microtubule dynamics (Trivedi et al., 2005).

## Roles of Axin in Axon Formation and Outgrowth

After neurons reach their final destinations, the leading process develops into an apical dendrite and extends up to the pia, while the trailing process differentiates into an axon and grows toward the intermediate zone. Over time, the apical dendrite branches extensively and is accompanied by basal dendrites arborizing radially from the cell soma (Whitford et al., 2002). The axons pass vertically through the cortical plate and intermediate zone, sprout collaterals that arborize in specific intracortical layers, and bundle to form axonal tracts that project to intra- and subcortical areas (Hatanaka and Murakami, 2002). The projection targets determine the three basic subtypes of neurons: associative, commissural, and corticofugal, in which axons form connections within the cortex in the same hemisphere, opposite hemisphere, or away from the cortex, respectively (Molyneaux et al., 2007). Neurons in distinctive cortical and subcortical areas are functionally coordinated and integrated through their axonal projections to allow the proper cortical information processing. The patterns of axon projection and connection involve axon formation and extension, guidance, recognition of and targeting to specific areas, and elimination of inappropriate axon segments and branches. Among these processes, axon formation and extension are the most basic and are regulated by cytoskeletal reorganization.

Axon is initiated and facilitated by the extracellular cues. For example, TGF-β and its receptors specify the axon during brain development. TGF-β receptors are expressed in axons during embryonic development, and their receptor kinase activity is required for axon formation (Yi et al., 2010). The effect of TGF-β signaling on axon specification is mediated by the phosphorylation of Par6, a component of the Par3/Par6/aPKC complex. Neurotrophic factors such as brain-derived neurotrophic factor (BDNF) can also direct axon specification, because the first neurite contacting a BDNF stripe becomes the axon (Shelly et al., 2007). The effect of BDNF on axon specification requires the activation of the polarity-inducing kinase LKB1 via a cAMP-dependent protein kinase A pathway (Shelly et al., 2007). In another case, both BDNF and neurotrophin 3 stimulate the inhibition of GSK3β, resulting in the dephosphorylation of CRMP2, which consequently promotes axon outgrowth in cultured hippocampal neurons (Yoshimura et al., 2005).

**Figure 5:**
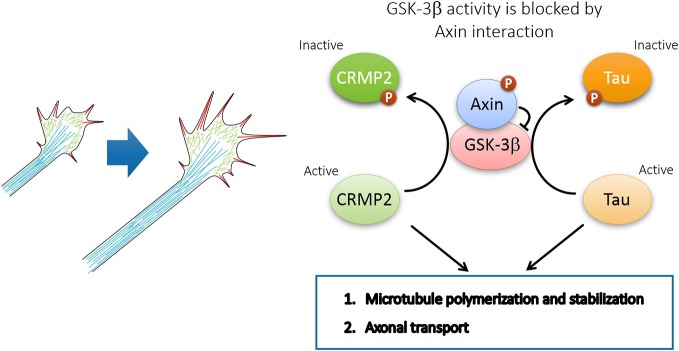
**Axin-GSK3β signaling promotes axon growth through microtubule assembly and axonal transport**. GSK3β inactivation by Axin interaction maintains CRMP2 and Tau in active form and coordinates the local axon assembly at the growth cone and efficient axon growth.

Interestingly, recent evidence demonstrates that Axin plays an instrumental role in microtubule assembly and axonal transport in supporting axon formation and growth. Through Axin knockdown *in utero*, our laboratory demonstrated that the Cdk5-mediated phosphorylation of Axin contributes to axon formation through the inhibition of GSK3β *in vivo* (Fang et al., 2011). In particular, Cdk5 is activated through p35 to phosphorylate Axin at Thr485 in response to neurotrophins such as BDNF and neurotrophin 3 (NT-3). The phosphorylation of Axin enhances its interaction with GSK3β, which inhibits GSK3β activity, thereby increasing nonphosphorylated CRMP-2 and Tau in the growth cone (Fang et al., 2011). Non-phosphorylated CRMP-2 and Tau, which represents the active form, promotes microtubule assembly and stabilization to support the elongation of growing axons during development. Therefore, Axin expression and phosphorylation by Cdk5 are essential for axon formation and outgrowth through the regulation of microtubule dynamics (Figure 5). In addition to microtubule dynamics, efficient axonal transport requires Cdk5-mediated suppression of GSK3β, thereby preventing premature GSK3β-mediated cargo release (Morfini et al., 2004). Whether Cdk5 inhibits GSK3β by facilitating Axin-GSK3β interaction awaits further investigation.

## Implications of Axin in Neurodevelopmental Disorders

As Axin is a multifaceted scaffold protein involved in diverse signaling pathways that regulate neural progenitor proliferation and differentiation, it is not surprising that its deregulation is associated with brain malformations such as micro- and macrocephaly. Notably, a previous study demonstrates that a mutation in the GSK3-binding domain of Axin results in the formation of small brains, mimicking human microcephaly (Heisenberg et al., 2001). Additional evidence in a human genetic study shows that the *Axin* gene is located in the same chromosomal region (16p13.3–12.1) as genetic mutations found in microcephaly patients (Kavaslar et al., 2000). Despite the lack of an Axin genetic mouse model, the recent development of *in utero* electroporation techniques have enabled the manipulation of Axin protein in neural progenitor cells during cerebral cortical development *in vivo* (Saito and Nakatsuji, 2001; Tabata and Nakajima, 2001). Accordingly, depletion or overexpression of Axin in the mouse neocortex results in premature and suppressed neuronal differentiation, which eventually lead to reduced and excessive numbers of cortical neurons at birth, respectively (Fang et al., 2013).

Axin is also implicated in the etiology of autism, a childhood-onset neurodevelopmental disorder characterized by disabilities in social interaction and communication as well as repetitive and compulsive behaviors. A postmortem study suggests that patients with autism often have increased neuronal density and number in the prefrontal cortex (McCaffery and Deutsch, 2005; Courchesne et al., 2011). We have recently generated a mouse model with enhanced neurogenesis by intraventricular injection of a tankyrase inhibitor, XAV939 (Figure 1; Huang et al., 2009). Injection of XAV939 transiently elevates the protein level of Axin in neural progenitors by preventing tankyrase-mediated protein degradation and consequently increases the number of upper-layer neurons in the developing mouse cortex without affecting astrogenesis or microglial reactivity (Fang et al., 2014). We demonstrated that enhanced neurogenesis leads to the overproduction of excitatory neurons, and impairs excitatory and inhibitory synaptic connection and balance. More importantly, these mice exhibit autism-like features, namely social interaction deficits in the three-chamber sociability test and compulsive behaviors such as increased self-grooming and marble burying (Fang et al., 2014). These results collectively illustrate a neurodevelopmental mechanism wherein enhanced neurogenesis and increased neuronal production result in autism-like features, hinting at the etiology of autism.

## Concluding Remarks and Perspectives

Current evidence from several studies clearly indicates that Axin acts as a scaffold protein to integrate upstream signals while regulating various downstream interacting proteins involved in a wide array of cellular activities. Specifically, cytosolic Axin modulates downstream signaling of extracellular signals, including neurotrophins, WNT, Notch, and TGFβ. In addition, Axin enables efficient cytoskeletal reorganization and axon transport through GSK3β signaling and various MAPs. Moreover, nuclear Axin regulates gene expression by interacting with β-catenin and activating various neurogenic transcriptional factors. Therefore, Axin appears to be a master scaffold that modulates various developmental steps in the development of cerebral cortex.

After embryonic development, lifelong neurogenesis occurs in specific regions of the adult brain; various Axin-regulated signaling proteins such as GSK3β and β-catenin continue to play important roles in adult neurogenesis (Lie et al., 2005; Mao et al., 2009; Morales-Garcia et al., 2012). Therefore, it will be of interest to investigate how Axin regulates the proliferation and differentiation of adult neural stem cells and if enhancing Axin protein level improves adult neurogenesis, which is implicated in depression and neurodegenerative diseases such as Alzheimer’s disease.

## Conflict of Interest Statement

The authors declare that the research was conducted in the absence of any commercial or financial relationships that could be construed as a potential conflict of interest.
